# An improved classification of G-protein-coupled receptors using sequence-derived features

**DOI:** 10.1186/1471-2105-11-420

**Published:** 2010-08-09

**Authors:** Zhen-Ling Peng, Jian-Yi Yang, Xin Chen

**Affiliations:** 1 Department of Electrical and Computer Engineering, University of Alberta, Edmonton, Alberta, T6G 2V4, Canada; 2 Division of Mathematical Sciences, School of Physical and Mathematical Sciences, Nanyang Technological University, 21 Nanyang Link, 637371, Singapore; 3 Department of Mathematics, Bijie University, Guizhou, 551700, China

## Abstract

**Background:**

G-protein-coupled receptors (GPCRs) play a key role in diverse physiological processes and are the targets of almost two-thirds of the marketed drugs. The 3 D structures of GPCRs are largely unavailable; however, a large number of GPCR primary sequences are known. To facilitate the identification and characterization of novel receptors, it is therefore very valuable to develop a computational method to accurately predict GPCRs from the protein primary sequences.

**Results:**

We propose a new method called PCA-GPCR, to predict GPCRs using a comprehensive set of 1497 sequence-derived features. The *principal component analysis *is first employed to reduce the dimension of the feature space to 32. Then, the resulting 32-dimensional feature vectors are fed into a simple yet powerful classification algorithm, called intimate sorting, to predict GPCRs at *five *levels. The prediction at the first level determines whether a protein is a GPCR or a non-GPCR. If it is predicted to be a GPCR, then it will be further predicted into certain *family*, *subfamily*, *sub-subfamily *and *subtype *by the classifiers at the second, third, fourth, and fifth levels, respectively. To train the classifiers applied at five levels, a non-redundant dataset is carefully constructed, which contains 3178, 1589, 4772, 4924, and 2741 protein sequences at the respective levels. Jackknife tests on this training dataset show that the overall accuracies of PCA-GPCR at five levels (from the first to the fifth) can achieve up to 99.5%, 88.8%, 80.47%, 80.3%, and 92.34%, respectively. We further perform predictions on a dataset of 1238 GPCRs at the second level, and on another two datasets of 167 and 566 GPCRs respectively at the fourth level. The overall prediction accuracies of our method are consistently higher than those of the existing methods to be compared.

**Conclusions:**

The comprehensive set of 1497 features is believed to be capable of capturing information about amino acid composition, sequence order as well as various physicochemical properties of proteins. Therefore, high accuracies are achieved when predicting GPCRs at all the five levels with our proposed method.

## Background

The structure of a G-protein-coupled receptor (GPCR) generally comprises seven *α*-helical transmembrane domains, an extracellular N-terminus, and an intracellular C-terminus [1]. GPCRs constitute one of the largest family of membrane proteins, and their main function is to transduce extracellular signals into intracellular reactions. Therefore, they play a key role in diverse physiological processes such as neurotransmission, secretion, cellular differentiation, cellular metabolism, and so forth [2]. It has been estimated that almost two-thirds of drugs on the market interact with GPCRs [3], which indicates that GPCRs are pharmacologically important. Therefore, both academic and industrial researchers are very interested in the studies on GPCRs to understand their structures and functions. Unfortunately, the 3 D protein structures of GPCRs are largely unavailable [4], except for the GPCR family *bovine rhodopsin*. Although some advanced biotechnologies such as NMR allow to detect the 3 D protein structures, their experiments are generally very time-consuming and costly. In contrast, a large number of GPCR primary sequences are known [5]. To facilitate the identification and characterization of novel receptors [5], it is therefore very valuable to develop a computational method to predict GPCRs from the protein primary sequences.

**Figure 1:**
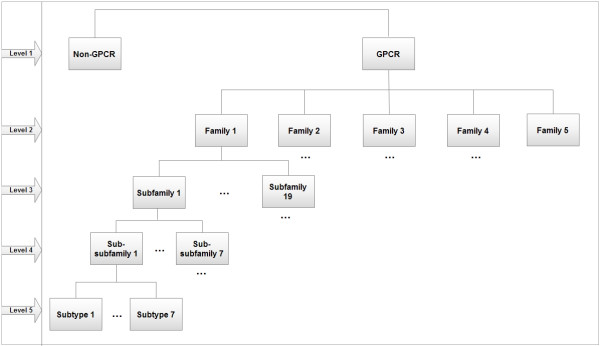
**The hierarchical structure for GPCRs**. The organization of GPCR sequences in the GPCRDB database does not include the first level in this figure. We add it in this study because we performed prediction at this level.

Based on their binding ligand types, GPCRs are often classified into different groups, some of which are further divided into subgroups, sub-subgroups, etc. The GPCRDB database [1,6] is one of the most popular database for GPCRs, which organizes GPCRs using a hierarchical structure. As in [7,8], we call each layer of this hierarchical structure a *level*. The top layer is then referred to as the second level (One more layer will be added on the top of the hierarchical structure later), and the second layer is referred to as the third level, etc. According to the latest version of the GPCRDB database (Version 9.9.1, September 2009), GPCRs in the second level are classified into five *families *or *classes *(In the previous versions of the GPCRDB database, e.g., June 2006 release, GPCRs are classified into six families in this level); that is, (1) *Class A Rhodopsin like*, (2) *Class B Secretin like*, (3) *Class C Metabotropic glutamate*/*pheromone*, (4) *Vomeronasal receptors *(*V1R *&*V3R*), and (5) *Taste receptors T2R*. For the first four families above, each is further divided into *subfamilies *located at the third level. Furthermore, located on the fourth and fifth levels of the hierarchical structure are the *sub-subfamilies *and *subtypes*, respectively. On the other hand, given a new protein, the first step is to determine whether it is a GPCR or a non-GPCR. Therefore, we add one more level on the top of the hierarchical structure of the above classification system. It is referred to as the *first *level. The complete hierarchical structure of five levels is illustrated in Figure 1.

In this paper we will look into the following classification problem, which is referred to as a *five-level *classification problem. Given a protein sequence, we need to determine whether it is a GPCR or a non-GPCR. If it is predicted into a GPCR, we need to further determine which family, subfamily, sub-subfamily, and subtype it belongs to. To tackle this problem, a set of distinct classifiers is generally needed for each level as depicted in Figure 1. In the literature, many computational methods have been proposed to predict GPCRs. However, to our best knowledge, there are no methods that can deal with the five-level problem completely, (i.e., allow to make predictions at all the five levels). For example, the methods presented in [9-12] predict GPCRs just at a single level (the second, third or fourth level), and the methods in [13] predict GPCRs only at the third and fourth levels. The prediction methods in [8] and [7] instead considered three and four levels, respectively.

Today's academic and industrial researchers are both interested in the functional roles of GPCRs at the finest *subtype *level. This is mainly because each subtype demonstrates its own characteristic ligand binding property, coupling partners of trimeric G-proteins, and interaction partners of oligomerization [14]. Therefore, discrimination of functions of a GPCR subtype from the others (i.e., prediction of GPCRs at the fifth level as shown in Figure 1) becomes very important in the effort to decipher GPCRs. However, we can expect that it is a challenging task that shall not be easier than the prediction of GPCRs at any of the first four levels. Fortunately, more and more GPCR sequences are now being accumulated into the GPCRDB database, which makes it possible to accurately predict GPCRs at all the five levels. This is the main goal of our present study.

A lot of related work has been done previously. In general, there are two important components in a classification task -- one is feature extraction and the other is a classification algorithm. Feature extraction means how to extract features from protein sequences so that each protein is represented as a fixed-length numerical vector. Various methods have been proposed to extract information from protein sequence in the past decades (See eg., [15-19]). The commonly-used feature extraction methods are based on amino acid composition [9-11] and dipeptide composition [7,12,13,20,21], and more complicated ones include Chou's pseudo amino acid composition [15], the cellular automaton image approach [16], profile hidden Markov models [22], fast Fourier transform [23], wavelet-based time series analysis [24], and Fisher Score Vectors [25]. Once protein sequences are represented by numerical vectors, any general-purpose classification algorithms can be used for classification, for instance, covariant discriminant [9-11,16], nearest neighbor [7], bagging classification tree [13], and support vector machines [12,20,21,23-25].

In this paper, we focus on predicting GPCRs at the five levels. Five groups of descriptors are used to extract information from the amino acid sequences. These five groups are (1) *amino acid composition and dipeptide composition*, (2) *autocorrelation descriptors*, (3) *global descriptors*, (4) *sequence-order descriptors*, and (5) *Chou's pseudo amino acid composition descriptors*. These descriptors reflect various physicochemical properties of proteins and have been adopted to predict many other protein attributes, such as protein subcellular localization [19,26], outer membrane protein [27], nuclear receptors [28], and protein structural classes [17,18]. By combining these descriptors, a comprehensive set of 1497 features are calculated for each amino acid sequence. By applying the *principal component analysis *on a dataset, we then reduce them to a set of 32 features that could retain as much of the data variability as possible.

Finally, a simple yet powerful algorithm called *intimate sorting *is employed to predict GPCRs, and the experimental tests on the benchmark datasets show that the classifications can be improved. Jackknife test shows that the overall accuracies of the proposed method at the first, second, third, fourth, and fifth levels achieve up to 99.5%, 88.8%, 80.47%, 80.3%, and 92.34%, respectively. Comparisons with several existing methods show that the proposed method achieves higher prediction performance consistently.

## Results and Discussion

### Predicting GPCR at five levels

**Figure 2:**
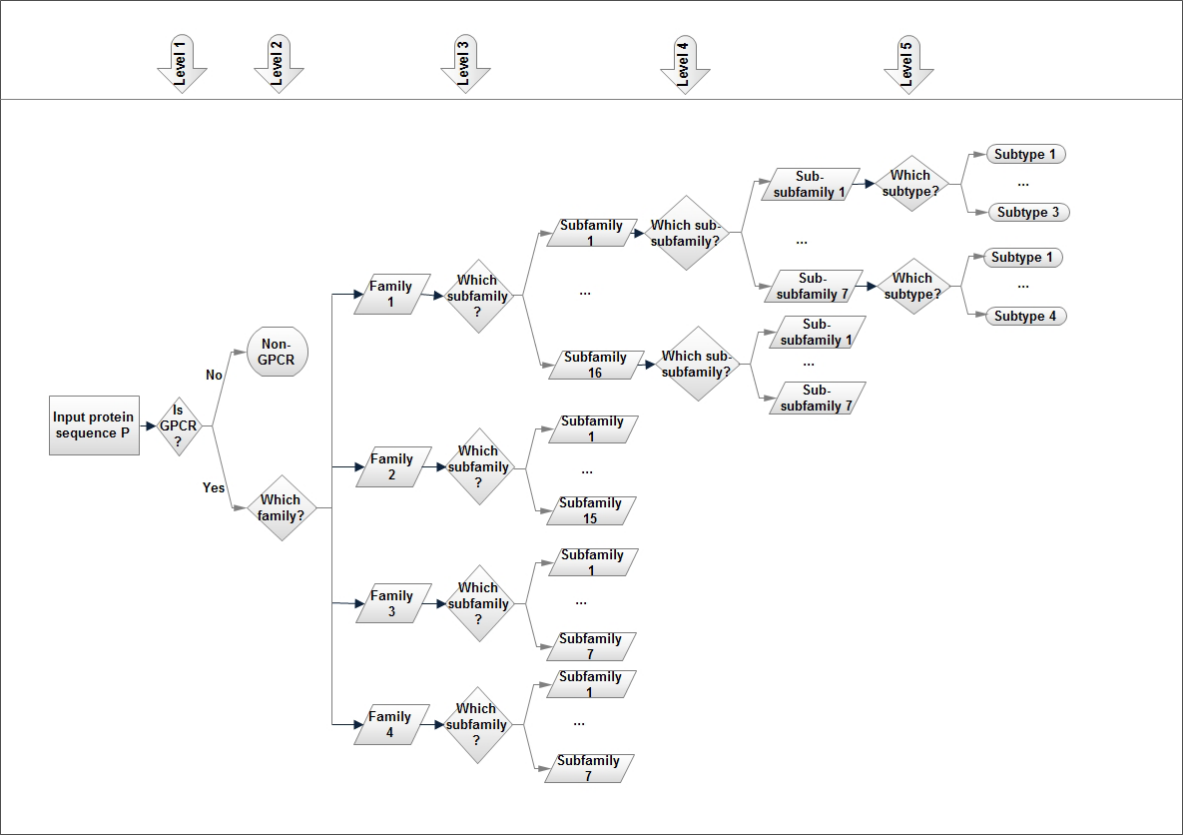
**The structure of PCA-GPCR**. For the name of the families, subfamilies, sub-subfamilies, and subtypes, please refer to the Additional file 1. The fourth and fifth levels are only applicable for some subfamilies and subtypes, which are also listed in the Additional file 1.

For simplicity, we call the proposed method PCA-GPCR. PCA-GPCR preforms the prediction at five levels, and its flowchart structure is depicted in Figure 2. By the first-level classifier a new protein sequence is predicted to be either a GPCR or a non-GPCR. If it is predicted to be a GPCR, it will be further classified into one of the four families, which is done by the second-level classifier. The third-level classifiers hence determine which subfamily the protein belongs to. For some subfamilies (see Additional file 1), the fourth-level classifiers are used to determine the sub-subfamily of the protein. Finally, the fifth-level classifiers determine the subtypes of the protein, if any (see Additional file 1). We carried out the experiments on the collection of datasets *GDFL *(Please see the **Methods **section for the details of datasets). Jackknife tests show that the overall accuracies of PCA-GPCR are 99.5%, 88.8%, 80.47%, 80.3%, and 92.34% for the five levels, respectively. The details of experimental results are presented in the Additional file 1. It is commonly believed that, the smaller number of training sequences, the less reliable a classifier to be trained. Therefore, it is not surprising to see that the prediction accuracies are higher at the first and second levels and relatively lower at the third and fourth levels. On the other hand, to filter out high-homology sequences, we used CD-HIT with a less stringent threshold (0.9) for the fifth level than the one for any other levels, which results in a larger number of training sequences for the fifth level. This might partly explain why the accuracy achieved for the fifth level (subtype) is higher than those of the second, third and fourth levels. For the convenience of public use, a web server was already developed, which is freely available at http://www1.spms.ntu.edu.sg/~chenxin/PCA_GPCR.

### Comparison with BLAST-based classification

The most straightforward method for predicting GPCRs might be based on homology search by sequence alignment tools such as BLAST and PSI-BLAST [29]. A given GPCR sequence is hence predicted into the class to which its most similar GPCR sequence belongs. However, as the pairwise sequence similarities get lower, such an alignment-based method would rarely yield satisfactory predictions. For instance, when applied to the dataset *GDFL *for the prediction at the first level, the BLAST-based method achieved the overall accuracy of 74.58%, which is 14.92% lower than that from PCA-GPCR. Note that PCA-GPCR is instead an alignment-free method. The above experimental results therefore show that an alignment-free method is very promising in the high accurate prediction of GPCR classes.

### Comparison with previous methods

In order to demonstrate the superior performance of PCA-GPCR, we make comparisons with a number of previous methods. Depending on the predictive capability of previous methods, the comparisons are made at a single level and at the first two levels, as follows.

#### Comparison at a single level

Because many previous methods predicted GPCR at a single level [9-12], we also predict GPCR at just one level in order to compare with them fairly. Three benchmark datasets that contain a proportion of high-homology sequence pairs, *D167*, *D566 *and *D1238*, are used here (Please see **Methods **section for the details of these datasets). The first two datasets comprise GPCRs from the fourth level, and the last one is composed of GPCRs from the second level. The resulting prediction accuracies for these datasets are listed in Table 1. We can see that the overall accuracies for three datasets are all above 97%. To be specific, the overall accuracies of 98.2%, 97.88%, and 99.76% are achieved for the datasets *D167*, *D566*, and *D1238*, respectively. They are slightly higher than the accuracies reported in Refs. [7,9-13,21]. Indeed, the prediction accuracies for individual families or sub-subfamilies are all very high and, in some cases, have reached 100% or nearly 100%.

**Table 1:** The number of proteins in four datasets and the corresponding prediction accuracies.

Dataset	Family/sub-subfamily	**Tot**(***i***)	*c*(*i*)	ACC(%)
*D167*	Acetylcholine	31	31	100
	Adrenoceptor	44	44	100
	Dopamine	38	36	94.74
	Serotonin	54	53	98.15
	*Overall*	167	164	98.2
*D566*	Adrenoceptor	66	65	98.48
	Chemokine	92	90	97.83
	Dopamine	43	40	93.02
	Neuropeptide	31	30	96.77
	Olfactory	84	84	100
	Rhodopsin	183	180	98.36
	Serotonin	67	65	97.01
	*Overall*	566	554	97.88
*D1238*	Rhodopsin-like	1103	1102	99.91
	Secretin-like	84	83	98.81
	Metabotrophic/glutamate/pheromone	51	50	98.04
	*Overall*	1238	1235	99.76
*D365*	Rhodopsin-like	232	222	95.69
	Secretin-like	39	34	87.18
	Metabotrophic/glutamate/pheromone	44	39	88.64
	Fungal pheromone	23	22	95.65
	CAMP receptor	10	10	100
	Frizzled/smoothened	17	11	64.71
	*Overall*	365	338	92.6

Tot(*i*) is the number of sequences observed in class *i*, *c*(*i*) is the number of correctly predicted sequences of class *i*, and ACC is the prediction accuracy.

Because the dataset *D167 *has been widely used to test various methods, it is adopted here for further detailed comparisons with the other five methods [7,10,12,13,21]. The experimental results are presented in Table 2. It is evident from the table that our method achieved the highest overall prediction accuracy. Our method performs better than any other tested methods in the predictions of the GPCR sub-subfamilies except for the sub-subfamily *Serotonin*.

**Table 2:** Comparison with other methods at the fourth level based on the D167 dataset.

**Reference**	**Acetylcholine**	**Adrenoceptor**	**Dopamine**	**Serotonin**	**Overall**
	
[10]	67.74	88.64	81.58	88.89	83.23
[13]	90.3	86.4	78.9	79.6	83.2
[12]	93.6	100	92.1	98.2	96.4
[7]	93.3	100	94.7	100	97.6
[21]	96.7	100	92.1	**100**	97.6
This paper	**100**	**100**	94.74	98.15	**98.2**

The results of the other methods are taken directly from the corresponding references.

#### Comparison with GPCR-CA at the first two levels

We further compare our method with GPCR-CA [16] on the dataset *D365*, which comprises GPCRs from the second level. Unlike the datasets tested in the above subsection, *D365 *contains almost no high-homology sequence pairs. Note that the GPCR-CA is able to predict GPCRs at the first two levels.

The prediction accuracies of both GPCR-CA and PCA-GPCR at the first and second levels are listed in Table 3 and Table 4, respectively. At the first level, to distinguish GPCRs from non-GPCRs, our method achieves the overall accuracy of 95.21%, which is 3.57% higher than that of GPCR-CA. At the second level, the overall accuracy of our method improves over GPCR-CA by 9.04%. Meanwhile, according to the prediction accuracies of individual families, our method performs much better than GPCR-CA except for the *rhodopsin-like *family. It is also noticeable that a substantial improvement of 86.95% (= 95.65% -8.70%) has been made for the prediction of the *fungal pheromone *family (partly due to the small size of protein sequences in this family, as shown in Table 1).

**Table 3:** Comparison with GPCR-CA in identifying the GPCRs and non-GPCRs.

Protein type	**GPCR-CA **[16]	This paper
GPCR	92.33	**96.99**
Non-GPCR	90.96	**93.42**
*Overall*	91.64	**95.21**

The results of GPCR-CA are directly taken from the Ref. [16].

**Table 4:** Comparison with GPCR-CA for the dataset D365 in predicting GPCR families.

Family	GPCR-CA	This paper
Rhodopsin-like	**96.55**	95.69
Secretin-like	74.36	**87.18**
Metabotrophic/glutamate/pheromone	81.82	**88.64**
Fungal pheromone	8.70	**95.65**
CAMP receptor	60	**100**
Frizzled/smoothened	47.06	**64.71**
*Overall*	83.56	**92.60**

The results of GPCR-CA are directly taken from the Ref. [16].

GPCR-CA extracts 24 features, including 20 features from amino acid composition and four features from cellular automaton image [16]. While the last four features were reported to be able to reveal the protein's overall sequence patterns, only four features might not suffice to reveal overall sequence patterns completely. On the contrary, our method explores the amino acid sequences comprehensively to gain as much information from the protein primary sequences as possible. Both the amino acid composition and the dipeptide composition are utilized in our method and, moreover, the important sequence-order information and a variety of physicochemical properties of amino acids are carefully explored as well. We believe that it is this comprehensive set of features that lead our method to a higher prediction accuracy.

## Conclusions

In this paper, we have proposed a new method called PCA-GPCR to predict GPCRs at five levels. In this method, a comprehensive set of 1497 sequence-derived features are generated from five groups of descriptors -- that is, *amino acid composition and dipeptide composition*, *autocorrelation descriptors*, *global descriptors*, *sequence-order descriptors*, and *Chou's pseudo amino acid composition descriptors*. These features are able to capture the information about the amino acid composition, sequence order as well as various physicochemical properties of proteins. Because of the high dimensionality of the feature space, the principal component analysis is hence used to reduce the dimension from 1497 to 32. The resulting 32-dimensional feature vectors are finally fed into a simple yet powerful intimate sorting algorithm for the prediction of GPCRs at five levels.

By evaluating on the datasets constructed from the latest version of the GPCRDB database, the overall accuracies of our method from the first level to the fifth level are 99.5%, 88.8%, 80.47%, 80.3%, and 92.34%, respectively. We further test and compare our method with several other methods based on four benchmark datasets widely used in the literature. At the second level, for a dataset containing 1238 GPCRs, the overall accuracy of our method reaches 99.76%. At the fourth level, for two different datasets that contain 167 and 566 GPCRs, the overall accuracies of our method reach up to 98.2% and 97.88%, respectively. They are all higher than those of the other methods under comparison. At the first two levels, we further test our method on a low-homology dataset (with only a few sequence pairs of more than 40% sequence identity). The overall accuracies thus achieved at the first level and second level are 95.21%, 92.6%, respectively, which are 3.57% and 9.04% higher than those of the method GPCR-CA.

We conclude that the high prediction accuracy of the proposed method is attributed to the comprehensive set of features that we constructed from five groups of descriptors. It is anticipated that our method could contribute more to the characterization of novel proteins and gain new insights into their functions, thereby facilitating drug discovery. A web server that predicts GPCRs at five levels with our proposed method is freely available at http://www1.spms.ntu.edu.sg/~chenxin/PCA_GPCR.

## Methods

### Datasets

We construct a collection of non-redundant datasets from the latest release of the GPCRDB database (Version 9.9.1, September 2009) [6] to evaluate and train the classifiers for the GPCRs prediction. As mentioned in the **Background **section, the sequences in the GPCRDB database are organized in four levels: family or class, subfamily, sub-subfamily, and subtype. We download the GPCR sequences from the GPCRDB database and then filter out the high-homology sequences using the program CD-HIT [30]. In order to ensure that there are enough sequences to train the classifiers, we apply different thresholds in CD-HIT for sequences at different levels. They are 0.4, 0.7, 0.8, and 0.9 for the family, subfamily, sub-subfamily, and subtype levels, respectively. After filtering, only families (subfamilies, sub-subfamilies, and subtypes) with more than 10 sequences are retained for training classifiers. Because the fifth family (*Taste receptors T2R*) has no subfamily and there are only 14 sequences remaining after filtering by CD-HIT, it is therefore ignored in subsequent analysis. At the end, we obtained 1589, 4772, 4924, and 2741 GPCRs at the family, subfamily, sub-subfamily and subtype levels, respectively. The name of families, subfamilies, sub-subfamilies, and subtype, together with the number of GPCR proteins retained at each level are listed in the Additional file 1.

The GPCR protein sequences retained at the family level are used to construct a positive dataset for training and evaluation. A negative dataset of non-GPCRs is then constructed in almost the same way as in Ref. [25], except that the latest version of ASTRAL SCOP (Version 1.75) [31] is used. First, we download the sequences that have less than 40% identity to each other (i.e., the file with the name "seq.75;item = seqs;cut = 40"). Then, remove those sequences of length less than 30, and those having identity above 40% using CD-HIT. Finally, a total of 10325 sequences remain, from which 1589 sequences are randomly selected to form a negative dataset. Because these selected proteins are organized into five levels, for the sake of convenience, we call them the datasets *GDFL *(GPCR Datasets in Five Levels). They are available at the web server provided in this paper.

In addition, in order to perform comparison with other existing methods directly, four benchmark datasets from previous studies are experimented in this study as well. For the sake of simplicity, they are referred to as *D167*, *D566*, *D1238 *and *D365*, respectively. We know that all of them were constructed based on the older version of the GPCRDB database. The proteins in the dataset *D167 *[10] (belonging to the fourth level) are classified into four sub-subfamilies: (1) *acetylcholine*, (2) *adrenoceptor*, (3) *dopamine*, and (4) *serotonin*. The dataset *D566 *[11] (belonging to the fourth level) instead comprises proteins in seven sub-subfamilies: (1) *adrenoceptor*, (2) *chemokine*, (3) *dopamine*, (4) *neuropeptide*, (5) *olfactory type*, (6) *rhodopsin*, and (7) *serotonin*. The dataset *D1238 *[9] (belonging to the second level) comprises proteins from three families: (1) *rhodopsin like*, (2) *secretin like*, and (3) *metabotrophic/glutamate/pheromone*. The last dataset *D365 *[16] (belonging to the second level) comprises proteins in the six families: (1) *rhodopsin-like*, (2) *secretin-like*, (3) *metabotrophic/glutamate/pheromone*; (4) *fungal pheromone*, (5) *cAMP receptor *and (6) *frizzled/smoothened family*. The numbers of proteins in the above four datasets are given in Table 1. Furthermore, 365 non-GPCR sequences are taken from the Swiss-Prot database to serve as a negative dataset against *D365 *[16].

The sequence homology level is an important factor that affects the effectiveness of a classification method. Therefore, it is worthwhile to take a look at the sequence similarity levels of proteins in these datasets before performing any evaluation test. For simplicity, we analyze the similarity level of the whole dataset rather than the subsets in the dataset. Chou and Elrod [9-11] reported that all the receptor sequences in the aforementioned datasets were generally lower than 40%, according to their definition of the *average sequence identity percentage *between two protein sequences. Here, we run a protein sequence clustering program called CD-HIT [30] on each dataset with the varying thresholds of sequence identity. For example, if a threshold of 0.9 is used, the proteins having pairwise residue identities of 90% or above would be placed into a same cluster. In general, the fewer resulting clusters imply the higher overall sequence similarities. The test results are shown in Table 5, where the proteins are clustered with the thresholds of 0.9, 0.8, 0.7, 0.6, 0.5 and 0.4, respectively. In particular, 100 clusters are obtained for 167 proteins in the dataset *D167 *with the threshold of 0.9. It indicates that there do exist high-homology protein pairs, but they only take up a small proportion of the total number (i.e., 12861 = 167 × 166/2) of distinct protein pairs. The use of the threshold of 0.4 further reduces the number of clusters to 30, which could suggest that the *average *sequence identity of proteins is quite low. However, to avoid the overestimation of prediction accuracy, it would be better if those high-homology sequences can be filtered out with CD-HIT. For instance, the dataset *D365 *does not contain any protein pairs having ≥ 40% pairwise sequence identity except in the E-cAMP receptor family, which contains too few (only 10) GPCRs to apply filtering.

**Table 5:** The CD-HIT clustering results for the four benchmark datasets.

		Dataset		
	
*γ*	*D167*	*D566*	*D1238*	*D365*
1.0	167	566	1238	365
0.9	100	346	777	361
0.8	73	226	540	361
0.7	61	169	421	361
0.6	52	142	358	359
0.5	38	106	281	357
0.4	30	69	207	356

*γ *denotes the threshold for the sequence identity percentage. The row of *γ *= 1.0 gives the total number of proteins in each dataset.

### Physicochemical properties

In order to capture as much information of protein sequences as possible, a variety of physicochemical properties [32] are used in the procedure of feature extraction. These physicochemical properties are listed in Table 6, of which the first eighteen are used to measure the physicochemical properties of individual amino acids and the last two to measure the physicochemical distances between two amino acids.

**Table 6:** The physicochemical properties of the amino acids and distances between two amino acids.

Order	Physicochemical property	Range of property	Reference
1	Hydrophobicity scales	[-1.14, 1.81]	[32]
2	Average flexibility indices	[0.295, 0.544]	[32]
3	Polarizability parameter	[0, 0.409]	[32]
4	Free energy of solution in water	[-2.24, 4.91]	[32]
5	Residue accessible surface area in tripeptide	[75, 255]	[32]
6	Residue volume	[36.3, 135.4]	[32]
7	Steric parameter	[0, 1.02]	[32]
8	Relative mutability	[18, 134]	[32]
9	Hydrophobicity	[-2.53, 1.38]	[33]
10	Hydrophilicity	[-3.4, 3]	[33]
11	Side-chain mass	[1, 130]	[33]
12	Normalized van der Waals volume	[0, 8.08]	[34]
13	Polarity	[4.9, 13.0]	[34]
14	Polarizability	[0, 0.409]	[34]
15	Charge	Positive, Neutral, Negative	[34]
16	Secondary structure	Helix, Strand, Coil	[34]
17	Solvent accessibility	Buried, Exposed, Intermediate	[34]
18	Relative hydrophobicity	Polar, Neutral, Hydrophobic	[34]
19	Grantham chemical distance	[0, 215]	[34]
20	Schneider-Wrede physicochemical distance	[0, 1]	[19]

### Sequence-derived features

As mentioned in the introduction, amino acid composition was widely used to transform GPCR sequences into 20-dimension numerical vectors [9-11]. However, the sequence order information would be completely lost. In order to address this issue, dipeptide composition was proposed to represent GPCR sequences by 400-dimension vectors, which captures local-order information and has been reported to improve classifications [7,12,13,20,21]. Recently, GPCR-CA [16] utilized the conception of *Chou's pseudo amino acid composition *[33] to represent each protein sequence by 24 features. The first 20 features correspond to the amino acid composition and the remaining four features are calculated from a so-called *cellular automation image*. These four features were shown capable of reflecting a protein's overall sequence pattern. Inspired by this work, we seek a new set of features that can comprehend as much information as possible from GPCR sequences. To this end, we investigate the following five groups of features, where the parameters are set to the same values as in [34].

#### Amino acid composition (AAC) and dipeptide composition (DC)

Amino acid composition is defined as the occurrence frequencies of 20 amino acids in a protein sequence.

That is,

(1) $$f_{A}(i)=\frac{n_{A}(i)}{L},$$

where each *i *= 1, 2, ⋯ , 20 corresponds to a distinct amino acid and *n*_*A*_(*i*) is the number of amino acid *i *occurring in the protein sequence of length *L*.

Similarly, dipeptide composition is defined as the occurrence frequencies of the 400 dipeptides (i.e., 400 amino acid pairs). That is,

(2) $$f_{D}(i)=\frac{n_{D}(i)}{L-1},$$

where each *i *= 1, 2, ⋯, 400 corresponds to one of the 400 dipeptides and *n*_*D*_(*i*) is the number of dipeptide *i *occurring in the sequence.

#### Autocorrelation descriptors (AD)

We use three autocorrelation descriptors -- *normalized Moreau-Broto autocorrelation descriptors*, *Moran autocorrelation descriptors *and *Geary autocorrelation descriptors*. They are all defined based on the value distributions of the first eight physicochemical properties of amino acids along a protein sequence (see Table 6). The measurement values of these properties are first standardized before we proceed to calculate the three autocorrelation descriptors. The standardization is performed as follows.

(3) $$\begin{array}{cc} P(i)=\frac{P_{0}(i)-{\bar{P}}_{0}}{\sigma }, & i=1,2,\cdots ,20, \end{array}$$

where *P*_0_(*i*) are the property value of the amino acid *i*, ${\bar{P}}_{0}=\frac{1}{20}\sum_{i=1}^{20}P_{0}(i)$, and $\sigma =\sqrt{\frac{1}{20}\overset{20}{\underset{i=1}{\sum }}{\left(P_{0}(i)-{\bar{P}}_{0}\right)}^{2}}$.

***Normalized Moreau-Broto autocorrelation descriptors ***are defined as:

(4) $$\begin{array}{cc} NMBA(d)=\frac{MBA(d)}{L-d}, & d=1,2,\cdots ,30, \end{array}$$

where $MBA(d)=\sum_{i=1}^{L-d}P(R_{i})P(R_{i+d}),R_{i}$ and *R*_*i + d *_are the amino acids at position *i *and *i *+ *d *along the protein sequence, respectively. As mentioned earlier, we use the same parameter values as in [34], so the maximum value of *d *is 30.

***Moran autocorrelation descriptors ***are defined as:

(5) $$\begin{array}{cc} MA(d)=\frac{\frac{1}{L-d}\sum_{i=1}^{L-d}(P(R_{i})-\tilde{P})(P(R_{i+d})-\tilde{P})}{\frac{1}{L}\sum_{i=1}^{L}({P(R_{i})-\tilde{P})}^{2}}, & d=1,2,\cdots ,30, \end{array}$$

where $\tilde{P}=\frac{1}{L}\sum_{i=1}^{L}P(R_{i})$ is the average value of the property of interest along the sequence.

***Geary autocorrelation descriptors ***are defined as:

(6) $$\begin{array}{cc} GA(d)=\frac{\frac{1}{2(L-d)}\sum_{i=1}^{L-d}{\left(P(R_{i})-P(R_{i+d})\right)}^{2}}{\frac{1}{L-1}\sum_{i=1}^{L}{\left(P(R_{i})-\tilde{P}\right)}^{2}}, & d=1,2,\cdots ,30. \end{array}$$

For each autocorrelation descriptor, we obtain 240 (= 30 × 8) features. In total, 720 (= 240 × 3) features will be obtained to describe a protein sequence.

#### Global descriptors (GD)

These descriptors were first proposed by Dubchak et al. [35] to predict protein folding classes, and later applied to predict human Pol II promoter sequences [36]. They are constructed as follows. Firstly, given each of the following seven amino acid properties: *normalized van der Waals volume*, *polarity*, *polarizability*, *charge*, *secondary structure*, *solvent accessibility and relative hydrophobicity *(i.e., properties 12-18 listed in Table 6), the 20 amino acids are divided into three groups according to their property values. Then, for a given amino acid sequence, we may obtain a new sequence of three symbols, each corresponding to one group of amino acids. Finally, three groups of quantities are defined on the new sequence; that is, composition (*Comp*), transition (*Tran*) and distribution (*Dist*), as demonstrated below.

For the sake of simplicity, suppose that a sequence is made of only two letters (A and B). *Comp *is defined as the occurrence frequency of each letter in the sequence. For example, we have a sequence BABBABABBABBAABABABBAAABBABABA, in which there are 14 As and 16 Bs. Therefore, the occurrence frequencies of A and B are 14/(14 + 16) × 100.00 = 46.67 and 16/(14 + 16) × 100.00 = 53.33, respectively. *Tran *is used to represent the occurrence frequency of pairs AB or BA. In the above sequence, there are 21 transitions from one letter to another, so *Tran *is computed as (21/29) × 100.00 = 72.14. On the other hand, *Dist *calculates the relative positions of the first, 25%, 50%, 75% and 100% of the total amount of a particular letter in the sequence. In the above sequence, for example, the first, 25%, 50%, 75% and 100% of the total amount of the letter B are located at the first, 6th, 12th, 20th and 29th positions, respectively. The quantities *Dist *for the letter B are hence 1/30 × 100.00 = 3.33, 6/30 × 100.00 = 20.00, 12/30 × 100.00 = 40.00, 20/30 × 100.00 = 66.67 and 29/30 × 100.00 = 96.67. Similarly, we can find the *Dist *values for the letter A; they are 6.67, 23.33, 53.33, 73.33 and 100.00. At the end, the global descriptors of the above sequence become

(*Comp*;*Tran*;*Dist*) = (46.67, 53.33; 72.14; 6.67, 23.33, 53.33, 73.33, 100.00, 3.33, 20.00, 40.00, 66.67, 96.67)

Suppose there are *n *distinct symbols in a sequence, then the number of features in *Comp*, *Tran*, and *Dist *are $\left(\begin{array}{l} n\\ 1 \end{array}\right)$, $\left(\begin{array}{l} n\\ 2 \end{array}\right)$, and 5 × *n*, respectively. Recall that the 20 amino acids are divided into three groups by each amino acid property, which leads to a new sequence of three symbols (*n *= 3). Following the similar procedure demonstrated above, we will obtain $21\left(=\left(\begin{array}{l} 3\\ 1 \end{array}\right)+\left(\begin{array}{l} 3\\ 2 \end{array}\right)+5\times 3\right)$ features to describe the new sequence (of three symbols). Combining all the features to be extracted based on the seven amino acid properties, we will obtain a total of 147 (= 21 × 7) features for each input protein sequence from the global descriptors.

#### Sequence-order descriptors (SD)

In order to derive sequence-order descriptors, we rely on two distance measures for amino acid pairs. One is called the *Grantham chemical distance matrix *[34], and the other called the *Schneider-Wrede physicochemical distance matrix *[19]. Then, the *jth-rank sequence-order-coupling number *is defined as:

(7) $$\begin{array}{cc} \tau (j)=\sum_{i=1}^{L-j}({d(R_{i},R_{i+j}))}^{2}, & j=1,2,\cdots ,30, \end{array}$$

where *d*(*R*_*i*_, *R*_*i +j*_) is one of the above distances between the two amino acids *R*_*i *_and *R*_*i + j *_located at position *i *and *i *+ *j*, respectively.

The *quasi-sequence-order descriptors *are defined as:

(8) $$QSO(i)=\{\begin{array}{cc} \frac{f_{A}(i)}{\sum_{j=1}^{20}f_{A}(j)+\omega \sum_{j=1}^{30}\tau (j)}, & \left(1\leq i\leq 20\right)\\ \frac{\omega \cdot \tau (i)}{\sum_{j=1}^{20}f_{A}(j)+\omega \sum_{j=1}^{30}\tau (j)}, & \left(21\leq i\leq 50\right) \end{array}$$

where *ω *is a weighting factor (default *ω *= 0.1).

We end up with 60 (= 30 × 2) sequence-order-coupling numbers and 100 (= 50 × 2) quasi-sequence-order descriptors. In total, there are 160 features extracted from the sequence-order descriptors.

#### Chou'is pseudo amino acid composition descriptors (PseAAC)

This set of features were originally developed by Chou [33] and have been used widely to predict various attributes of proteins, such as outer membrane protein [27], nuclear receptors [28], and protein structural classes [17,18]. The Chou's pseudo amino acid composition descriptors are defined similarly as the quasi-sequence-order descriptors. The difference lies in the coupling number *τ *(*j*), which is modified to:

(9) $$\begin{array}{cc} \theta (d)=\frac{1}{L-d}\sum_{i=1}^{L-d}\Theta (R_{i},R_{i+d}), & d=1,2,\cdots ,30, \end{array}$$

where Θ (*R*_*i*_, *R*_*i + d*_) is the *d*th-tier correlation factor that reflects the sequence order correlation between all the most contiguous residues along a protein chain. It is defined as:

(10) $$\Theta (R_{i},R_{i+d})=\frac{1}{3}\sum_{k=1}^{3}{\left[H_{k}(R_{i})-H_{k}(R_{i+d})\right]}^{2},$$

where *H*_1_(*R*_*i*_), *H*_2_(*R*_*i*_) and *H*_3_(*R*_*i*_) are the *hydrophobicity*, *hydrophilicity*, and *side-chain mass of amino acid*, respectively [33]. Their original values are standardized in the same way as we have done in the definition of autocorrelation descriptors (i.e., eq. (3)). Finally, the Chou'is pseudo amino acid composition descriptors are defined as:

(11) $$PseAAC(i)=\{\begin{array}{cc} \frac{f_{A}(i)}{\sum_{j=1}^{20}f_{A}(j)+\omega \sum_{d=1}^{30}\theta (d)}, & \left(1\leq i\leq 20\right)\\ \frac{\omega \cdot \theta (i)}{\sum_{j=1}^{20}f_{A}(j)+\omega \sum_{d=1}^{30}\theta (d)}, & \left(21\leq i\leq 50\right) \end{array}$$

where *ω *is a weighting factor (default *ω *= 0.1). It will generate 50 features from the Chou'is pseudo amino acid composition descriptors.

In summary, a comprehensive set of 1497 features, which measure the protein sequences from different aspects, will be generated from the above five descriptors. The number of features in each group of descriptors is listed in Table 7. These features are used to represent every protein sequence, and may be directly fed into a classification algorithm. Note that, however, there are some correlations or redundancies among these features such as the first twenty features in the fourth and fifth groups of features. On the other hand, the dimension of the features is too large, which might make it difficult to work with many machine learning algorithms for classification. Therefore, it is necessary to reduce the dimension. In this study, we adopt one of the most popular and powerful techniques, namely, *principal component analysis*, for the purpose of dimensionality reduction.

**Table 7:** The number of features in each group of descriptors.

Order	Name	Number of features
(i)	Amino acid composition (AAC) and dipeptide composition (DC)	420
(ii)	Autocorrelation descriptors (AD)	720
(iii)	Global descriptors (GD)	147
(iv)	Sequence-order descriptors (SD)	160
(v)	Chou'is pseudo amino acid composition descriptors (PseAAC)	50
(vi)	All features	1497

### Principal component analysis

*Principal Component Analysis *(PCA) is a classical statistical method which is still widely used in modern data analysis. PCA involves a mathematical procedure that transforms a large number of (possibly) correlated variables into a smaller number of uncorrelated variables, *called principal components *(PCs), that retain as much variability of the data as possible [37]. Given a data matrix denoted by *X *= (*X*_1_, *X*_2_, ⋯, *X*_p_), where *X*_*i *_is a column vector of size *n *which is equal to the number of proteins of interest and *p *denotes the number of protein sequence features, a typical PCA is performed as follows.

First, we shall standardize every *X*_*i *_by

(12) $$\begin{array}{cc} Y_{i}=\frac{X_{i}-{\bar{X}}_{i}}{\sqrt{\text{Var}(X_{i})}}, & i=1,2,\cdots ,p, \end{array}$$

where ${\bar{X}}_{i}$ and Var(*X*_*i*_) are the mean and variance of the vector components of *X*_*i*_, respectively. Then, the covariance matrix of *Y *= (*Y*_1_, *Y*_2_, ⋯, *Y*_*p*_) is obtained as

(13) $$\text{Cov}(Y)=\frac{1}{n-1}Y^{T}Y.$$

For the covariance matrix Cov(*Y*), we find all its eigenvalues *λ*_1 _≥ *λ*_2 _≥ ⋯ ≥ *λ*_*p *_and the corresponding eigenvectors, *E*_1_, *E*_2_, ⋯, *E*_*p*_. Note that each *E*_*i *_= (*E*_*i*, 1_, *E*_i, 2_, ⋯, *E*_*i, p*_)^**T **^is a column vector of size *p *and *E*_1_, *E*_2 _⋯, *E*_*p *_are linearly uncorrelated according to the basic knowledge of linear algebra. Finally, we construct the *i*-th PC *PC*(*i*) as the linear combination of *Y*_1_, *Y*_2_, ⋯, *Y*_*p *_with the coefficients being the elements of the *i*-th eigenvector *E*_*i*_, i.e.,

(14) $$\begin{array}{cc} PC(i)=\sum_{j=1}^{p}E_{i,j}Y_{j}, & i=1,2,\cdots ,p. \end{array}$$

We can see that each *PC*(*i*) is a column vector with size *n *and the *j*-th element in *PC*(*i*) represents the *i*-th PC value of protein *j*. Thereafter, a total of *p *uncorrelated PCs are obtained.

In order to reduce the dimension of the feature space, only the first *m *PCs are used to represent each protein sequence (m ≤ *p*). It is generally hard to determine the optimal value of *m*. In this study, we aim to find a value of *m *that could make the overall prediction accuracy of GPCRs as high as possible, which we will further discuss later.

### Intimate sorting algorithm

Many classification algorithms in the literature have been used to predict GPCRs, for instance, covariant discriminant [9-11,16], nearest neighbor [7], bagging classification tree [13], and support vector machines [12,20,21,23-25]. In this study, we use a simple yet powerful algorithm called *intimate sorting *[26]. This algorithm is easy to implement and does not need to set any parameters as some other algorithms (e.g., support vector machines).

Suppose that a training set consists of *N *proteins {**P**_1_, **P**_2_, ⋯, **P**_*N*_}, each of which **P**_*i *_is a *λ*-dimension vector, **P**_*i *_= (*p*_*i*, 1_, *p*_*i*, 2_, ⋯, *p*_*i*_, *λ*)^**T**^. The GPCR classes of these proteins are already known, and each protein belongs to exactly one of the *μ *classes. The intimate sorting algorithm aims to place a query protein **P **= (*p*_1_, *p*_2_, ⋯, *p*_*λ*_)^**T **^into one of the *μ *classes based on the information from the *N *proteins in the training set. To this end, a measure of *similarity score *between **P **and **P**_*i *_is defined as

(15) $$\begin{array}{cc} \Phi (P,P_{i})=\frac{P\cdot P_{i}}{\left||P||||P_{i}|\right|}, & i=1,2,\cdots ,N, \end{array}$$

where $P\cdot P_{i}=\sum_{j=1}^{\lambda }p_{j}p_{i,j},\|P\|=\sqrt{\sum_{j=1}^{\lambda }p_{j}^{2}}$ and $\|P_{i}\|=\sqrt{\sum_{j=1}^{\lambda }p_{i,j}^{2}}$. When **P **≡ **P**_*i*_, it can be easily seen that Φ(**P**, **P**_*i*_) = 1, suggesting that they are most likely to belong to a same class. In general, we have -1 ≤ Φ(**P**, **P**_*i*_) ≤ 1. The higher the Φ(**P**, **P**_*i*_) value, the more likely two proteins belong to a same class. Among the *N *proteins in the training set, the one with the highest score with the query protein **P **is picked out, which we denote by **P**_*k*_, *k *∈ [1, *N*]. If there is a tie, we would randomly select one of them. In the final step, the intimate sorting algorithm simply assigns **P **into the same GPCR class as **P**_*k*_.

### Prediction assessment

The *jackknife test *is a rigorous and objective statistical test that can always yield a unique result for a given test dataset [38]. Therefore, it is often used to examine the power of a new classifier. In this paper, we also use it to evaluate our method, where proteins are singled out from the dataset one by one as a testing protein and the classifier is trained by the remaining proteins. In this sense, the jackknife test is also called the *leave-one-out *test. The prediction accuracies (*ACC*) and overall accuracy (*OACC*) are then measured by the following formulae:

(16) $$\begin{array}{cc} ACC(i)=\frac{C(i)}{\text{Tot}(i)}, & i=1,2,\cdots ,\mu , \end{array}$$

(17) $$\begin{array}{cc} OACC=\frac{\sum_{i}^{\mu }C(i)}{\sum_{i}^{\mu }\text{Tot}(i)}, & i=1,2,\cdots ,\mu , \end{array}$$

where Tot(*i*) is the total number of sequences in class *i*, *C*(*i*) the number of correctly predicted sequences of class *i*, and *μ *the total number of classes under consideration. Note that this prediction assessment method was already adopted in several previous studies, e.g., [7,15,16,24].

### Selection of *m*

**Figure 3:**
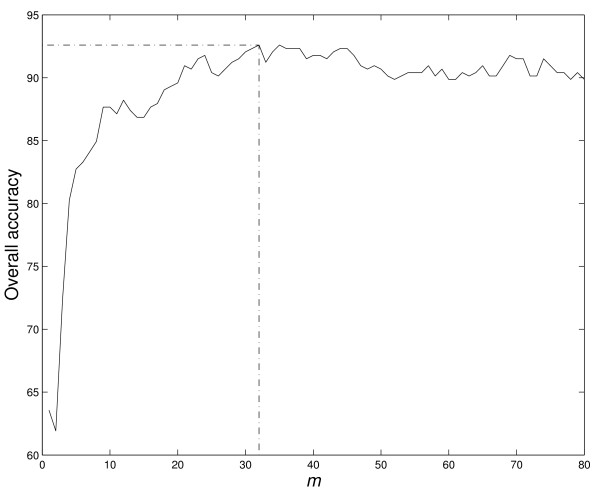
**Selection of *m***. The overall prediction accuracies of GPCR families for the *D365 *dataset obtained by varying the number *m *of principle components. The highest overall accuracy is achieved when *m *= 32, which is marked by the dotted lines.

As we mentioned earlier, the number of PCs in PCA, i.e., *m*, remains to be determined. Here, we choose its value by aiming to achieve the overall prediction accuracy as high as possible. To this end, we use the dataset *D365 *to compute the overall prediction accuracies *OACC*s of GPCR families for varying values of *m*. When *m *ranges from 1 to 80, *OACC*s thus obtained are plotted in Figure 3. We found that the highest accuracy (92.6%) is achieved with *m *= 32. Based on this observation, we chose *m *= 32 in our experiments.

### Contribution of features

Inspired by the PCA-based feature selection method described in [37,39], we use the following procedure to assess the contributions of the 1497 features to prediction accuracy. Recall that in the previous principle component analysis on the dataset *D365 *we obtained 32 eigenvectors *E*_1_, *E*_2_, ⋯, *E*_32_, and each eigenvector comprises 1497 components. Let us denote *E*_*i *_= (*E*_*ij*_), where 1 ≤ *i *≤ 32 and 1 ≤ *j *≤ 1497. To find the *i*-th PC in PCA, *E*_*ij *_is used to weight the *j*-th feature. In this sense, the value *E*_*ij *_can be viewed as the weight of contributions that the *j*-th feature makes to the *i*-th PC. To combine the contributions to all the PCs, we may compute $w_{j}=\sqrt{\sum_{i=1}^{32}E_{ij}^{2}}$. Then, *w*_*j *_can be naturally viewed as the weight of contributions that the *j*-th feature makes to the final prediction accuracy because our method is based on these 32 PCs. In general, the higher the weight *w*_*j*_, the more contributions the *j*-th feature makes.

**Figure 4:**
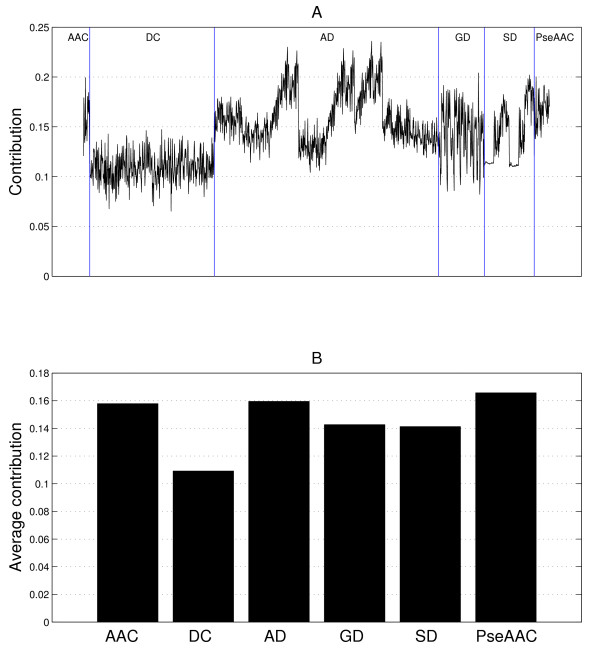
**Contribution of features**. The meanings of the notations AAC, DC, AD, GD, SD, and PseAAC can be found in Table 7. The divisions of these six subsets are marked by vertical blue lines.

The contribution of each of the 1497 features is computed and depicted in Figure 4(A), where we can see that the contributions of the amino acid composition (AAC) in the first group of descriptors are much higher than those of the dipeptide composition (DC). Therefore, we separate the AAC from the DC in the first group of descriptors in the following discussions. In addition, we find that the features from the autocorrelation descriptors (AD) made the highest contributions among all the features. Because there are 1497 features, it is not convenient to discuss the contributions of all individuals one by one. Instead, we compute the average contributions of the features in the following six subsets: AAC, DC, AD, GD, SD, and PseAAC. Their results are shown in Figure 4(B). It is evident from the figure that the highest average contribution is obtained with the features in the PseAAC subset (0.1657). The slightly lower contributions are provided by the AD and AAC subsets (0.1595 and 0.1579, respectively). On the contrary, the features in the GD and SD subsets achieve the average contributions only slightly higher than 0.14. The features in the DC subset instead achieve the least average contribution (0.1092). In summary, if we arrange the features in the six subsets in a decreasing order of their average contributions, then we obtain PseAAC, AD, AAC, GD, SD, and DC.

**Figure 5:**
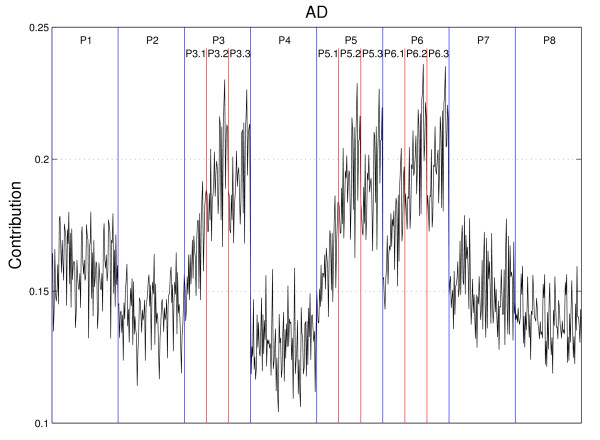
**Contribution of features in the AD subset**. The divisions of these eight groups are marked by vertical blue lines. Among the groups P3, P5 and P6, their divisions are marked by vertical red lines.

In particular, among the AD subset, some features made quite high contributions while the others made relatively low contributions, as we can see in Figure 4(A). For a thorough investigation, we plot the contributions of all the features in the AD subset again in Figure 5. These features are divided into eight groups according to the physicochemical properties used to compute them. In Figure 5, the eight groups of features are separated by vertical blue lines and indicated by P1, P2, ..., P8, respectively. Note that P*i *represents the *i*-th physicochemical property listed in Table 6. It is evident from Figure 5 that the highest contributions are due to the features computed with the physicochemical properties P3, P5 and P6; they are the polarizability parameter, residue accessible surface area in tripeptide and residue volume, respectively. For the group P3, we can further divide its 90 features into three subgroups according to three different autocorrelation descriptors (normalized Moreau-Broto, Moran, and Geary autocorrelation descriptors). These three subgroups are separated by red vertical lines in Figure 5, and indicated by P3.1, P3.2 and P3.3, respectively. In each subgroup, the feature contributions are computed with the values of *d *varying from 1 to 30 (from left to right on the horizontal axis). Observe that Moran and Geary autocorrelation descriptors (P3.2 and P3.3) made much higher contributions than the normalized Moreau-Broto descriptors (P3.1). Furthermore, for Moran and Geary autocorrelation descriptors, the features that are computed with a value of *d *in the range from 20 to 30 generally give rise to a fairly high contribution, while the maximum is attained when *d *= 26. The similar characteristics can also be observed for the groups P5 and P6 from Figure 5.

## Authors' contributions

ZLP and JYY contributed equally to the conception and design of the study, analyzed the results, have been involved in programming, drafting and revising the manuscript. XC has been involved in drafting and revising the manuscript. All authors wrote and approved the final manuscript.

## Supplementary Material

Additional file 1 **The information about families, subfamilies, sub-subfamilies, and subtypes**. The names of families, subfamilies, sub-subfamilies, and subtypes used by PCA-GPCR are listed in this file. The names are derived from GPCRDB database. The number of proteins in each family, subfamily, sub-subfamily, subtype, and the corresponding accuracies are also available in this file. Click here for file
