# Supplementary material for: An improved classification of G-protein-coupled receptors using sequence-derived features
Source: BMC Bioinformatics. 2010 Aug 9;11:420. doi: 10.1186/1471-2105-11-420 (PMC3247138; doi:10.1186/1471-2105-11-420)
Supplement: Additional file 1 — The information about families, subfamilies, sub-subfamilies, and subtypes. The names of families, subfamilies, sub-subfamilies, and subtypes used by PCA-GPCR are listed in this file. The names are derived from GPCRDB database. The number of proteins in each family, subfamily, sub-subfamily, subtype, and the corresponding accuracies are also available in this file. [file 1471-2105-11-420-S1.PDF]

Supplementary material for "An improved classification of G-protein-coupled receptors using sequence-derived features"

Zhen-Ling Peng, Jian-Yi Yang* and Xin Chen

The name of families, subfamilies, sub-subfamilies and subtypes in the GPCRDB database (http://www.gpcr.org/7tm/) and the corresponding prediction accuracies of PCA-GPCR.

The code of ***Subfamily*** and ***Sub-subfamily*** No. also represents the classification of the GPCR proteins. For example, the No. 001-002 means the ***Subfamily*** "***Peptide***" is the subfamily of the ***Family*** 001, i.e., "***Class A Rhodopsin like***". And the No. 001-002-003 means the ***Sub-subfamily*** "***Bradykinin***" is the subfamily of the ***Subfamily*** 001-002, i.e., "***Peptide***".

Tot(i) is the number of sequences observed in class *i*, *c(i)* is the number of correctly predicted sequences of class *i*, and ACC is an prediction accuracy.

Level 1 (**GPCR *Vs* Non-GPCR**, each sequence in the GPCR and Non-GPCR datasets has less than 40% identity with other sequences in the same dataset)

Name	*Tol(i)*	*C(i)*	*ACC (%)*
GPCR	1589	1589	100
Non-GPCR	1589	1581	99.75
Overall	3178	3170	99.5

Level 2 (**Family**, each sequence in a family has less than 40% identity with other sequences in the same family.)

Family No. (Name)	*Tol(i)*	*C(i)*	*ACC (%)*
001 (Class A Rhodopsin like)	1138	1058	92.97
002 (Class B Secretin like)	238	175	73.53
003 (Class C Metabotropic glutamate/pheromone)	168	144	85.71
004 (Vomeronasal receptors (V1R & V3R))	45	34	75.56
Overall	1589	1411	88.80

Level 3 (**Subfamily**, each sequence in a subfamily has less than 70% identity with other sequences in the same subfamily.)

Subfamily No. (Name)	*Tol(i)*	*C(i)*	*ACC (%)*
001-001 (Amine)	467	398	85.22
001-002 (Peptide)	1058	832	78.64
001-003 (Hormone protein)	53	48	90.57
001-004 ((Rhod)opsin)	189	138	73.02
001-005 (Olfactory)	1319	1295	98.18
001-006 (Prostanoid)	41	22	53.66
001-007 (Nucleotide-like)	153	86	56.21
001-008 (Gonadotropin-releasing hormone)	55	37	67.27
001-009 (Thyrotropin-releasing hormone & Secretagogue)	29	9	31.03
001-010 (Melatonin)	59	16	27.12
001-011 (Viral)	11	3	27.27
001-012 (Lysosphingolipid & LPA (EDG))	30	9	30.00
001-013 (Leukotriene B4 receptor)	15	4	26.67
001-014 (Ecdysis triggering hormone receptor)	12	7	58.33
001-015 (CAPA)	10	5	50.00
001-016 (Class A Orphan/other)	274	189	68.98
Overall	3775	3098	82.07
002-001 (Calcitonin)	30	27	90.00
002-002 (Corticotropin releasing factor)	16	6	37.50
002-003 (Gastric inhibitory peptide)	17	15	88.24
002-004 (Glucagon)	11	11	100.00
002-005 (Growth hormone-releasing hormone)	12	11	91.67
002-006 (Diuretic hormone)	13	13	100.00
002-007 (ERM1)	22	21	95.45
002-008 (Latrophilin)	38	31	81.58
002-009 (Brain-specific angiogenesis inhibitor (BAI)))	11	11	100.00
002-010 (Methuselah-like proteins (MTH))	42	42	100.00
002-011 (Cadherin EGF LAG (CELSR))	24	23	95.83
002-012 (GPR124)	10	9	90.00
002-013 (GPR64)	30	16	53.33
002-014 (GPR126)	15	6	40.00
002-015 (GPR133)	68	44	64.71
Overall	359	286	79.67
003-001 (Metabotropic glutamate)	96	70	72.92
003-002 (GABA-B)	87	84	96.55
003-003 (Vomeronasal)	36	26	72.22
003-004 (Phermone)	64	39	60.94
003-005 (Odorant)	31	28	90.32
003-006 (Taste)	18	17	94.44
003-007 (Class C Other)	48	23	47.92
Overall	380	287	75.53
004-001 (Vomeronasal receptors V1RD)	15	15	100.00
004-002 (Vomeronasal receptors V1RE)	18	17	94.44
004-003 (Vomeronasal receptors V1RF)	19	9	47.37
004-004 (Vomeronasal receptors V1RG)	50	28	56.00
004-005 (Vomeronasal receptors V1RH)	29	13	44.83
004-006 (Vomeronasal receptors V1RJ & VIRK)	43	38	88.37
004-007 (Vomeronasal receptors V1RL)	84	49	58.33
Overall	258	169	65.50
Overall (of all subfamilies)	4772	3840	80.47

Level 4 (**Sub-subfamily**, each sequence in a sub-subfamily has less than 80% identity with other sequences in the same sub-subfamily.)

Sub-subfamily No. (Name)	*Tol(i)*	*C(i)*	*ACC (%)*
001-001-001 (Muscarinic acetylcholine)	62	60	96.77
001-001-002 (Adrenoceptors)	122	107	87.70
001-001-003 (Dopamine)	107	85	79.44
001-001-004 (Histamine)	57	47	82.46
001-001-005 (Serotonin)	177	148	83.62
001-001-006 (Octopamine)	32	27	84.38
001-001-007 (Trace amine)	56	51	91.07
Overall	613	525	85.64
001-002-001 (Angiotensin)	20	15	75.00
001-002-002 (Bombesin)	40	29	72.50
001-002-003 (Bradykinin)	30	22	73.33
001-002-004 (Anaphylatoxin)	30	27	90.00
001-002-005 (Fmet-leu-phe)	58	48	82.76
001-002-006 (APJ like)	19	15	78.95
001-002-007 (Interleukin-8)	21	15	71.43
001-002-008 (Chemokine)	166	147	88.55
001-002-009 (Cholecystokinin CCK)	44	35	79.55
001-002-010 (Endothelin)	21	18	85.71
001-002-011 (Melanocortin)	41	38	92.68
001-002-012 (Prolactin-releasing peptide (GPR10))	31	22	70.97
001-002-013 (Neuropeptide Y)	252	205	81.35
001-002-014 (Neurotensin)	10	10	100.00
001-002-015 (Opioid)	24	22	91.67
001-002-016 (Somatostatin)	65	44	67.69
001-002-017 (Tachykinin)	72	60	83.33
001-002-018 (Vasopressin-like)	73	64	87.67
001-002-019 (Galanin like)	97	70	72.16
001-002-020 (Proteinase-activated like)	49	42	85.71
001-002-021 (Orexin and neuropeptides FF,QRFP)	12	2	16.67
001-002-022 (Urotensin II)	16	14	87.50
001-002-023 (GPR37 / endothelin B-like)	15	14	93.33
001-002-024 (Neuromedin U like)	52	40	76.92
001-002-025 (Somatostatin- and angiogenin-like peptide)	17	17	100.00
001-002-026 (Allatostatin C / drostatin C)	15	13	86.67
001-002-027 (Melanin-concentrating hormone receptors)	25	15	60.00
001-002-028 (Prokineticin receptors)	29	20	68.97
Overall	1344	1083	80.58
001-003-001 (Thyrotropin)	20	20	100.00
001-003-002 (Gonadotropin type I)	28	27	96.43
Overall	48	47	97.92
001-004-001 (Rhodopsin Vertebrate)	149	132	88.59
001-004-002 (Rhodopsin Cephalochordata)	11	9	81.82
001-004-003 (Rhodopsin Arthropod)	106	92	86.79
Overall	266	233	87.59
001-005-001 (Olfactory II fam 1 / MOR125-138,156)	41	29	70.73
001-005-002 (Olfactory II fam 2 / MOR256-262,270-285)	13	12	92.31
001-005-003 (Olfactory II fam 3 / MOR255)	58	41	70.69
001-005-004 (Olfactory II fam 4 / MOR225-248)	35	27	77.14
001-005-005 (Olfactory II fam 5 / MOR172-224,249,254)	17	16	94.12
001-005-006 (Olfactory II fam 6 / MOR103-105,107-119)	26	21	80.77

001-005-007 (Olfactory II fam 7 / MOR139-155)	11	7	63.64
001-005-008 (Olfactory II fam 8 / MOR161-171)	39	31	79.49
001-005-009 (Olfactory II fam 9 / MOR120)	16	7	43.75
001-005-010 (Olfactory II fam 10 / MOR263-269)	11	8	72.73
001-005-011 (Olfactory II fam 12 / MOR250)	38	24	63.16
001-005-012 (Olfactory II fam 13 / MOR253)	11	11	100.00
001-005-013 (Olfactory 14)	12	9	75.00
001-005-014 (Olfactory 15)	16	16	100.00
001-005-015 (Olfactory 16)	28	18	64.29
001-005-016 (Olfactory 17)	18	12	66.67
001-005-017 (Olfactory 18)	44	25	56.82
001-005-018 (Olfactory 19)	11	11	100.00
001-005-019 (Olfactory 20)	17	13	76.47
001-005-020 (Olfactory 24)	24	12	50.00
001-005-021 (Olfactory 25)	83	70	84.34
001-005-022 (Olfactory 31)	15	11	73.33
001-005-023 (Olfactory 37)	46	34	73.91
001-005-024 (Olfactory 38)	16	11	68.75
001-005-025 (Olfactory 47)	10	10	100.00
001-005-026 (Olfactory 50)	40	38	95.00
001-005-027 (Olfactory 58)	14	8	57.14
001-005-028 (Olfactory 59)	25	19	76.00
001-005-029 (Olfactory 60)	12	7	58.33
001-005-030 (Olfactory 64)	11	7	63.64
001-005-031 (Olfactory 71)	124	82	66.13
001-005-032 (Olfactory 73)	14	5	35.71
001-005-033 (Olfactory 76)	10	5	50.00
001-005-034 (Olfactory 79)	10	9	90.00
001-005-035 (Olfactory 86)	18	16	88.89
001-005-036 (Olfactory 89)	19	14	73.68
001-005-037 (Olfactory 101)	10	9	90.00
001-005-038 (Olfactory 102)	17	11	64.71
001-005-039 (Olfactory 106)	31	24	77.42
001-005-040 (Olfactory 115)	40	32	80.00
001-005-041 (Olfactory 127)	10	2	20.00
001-005-042 (Olfactory 129)	22	12	54.55
001-005-043 (Olfactory 132)	11	4	36.36
001-005-044 (Olfactory 134)	21	12	57.14
001-005-045 (Olfactory 135)	16	12	75.00
001-005-046 (Olfactory 136)	12	8	66.67
001-005-047 (Olfactory 138)	10	4	40.00
001-005-048 (Olfactory 147)	11	6	54.55
001-005-049 (Olfactory 150)	11	10	90.91
001-005-050 (Olfactory 183)	15	11	73.33
001-005-051 (Olfactory 209)	16	12	75.00
001-005-052 (Olfactory 210)	12	9	75.00
001-005-053 (Olfactory 214)	14	8	57.14
001-005-054 (Olfactory 215)	23	17	73.91
001-005-055 (Olfactory 216)	12	3	25.00
001-005-056 (Olfactory 224)	15	10	66.67
001-005-057 (Olfactory 225)	16	9	56.25
001-005-058 (Olfactory 226)	96	63	65.63
001-005-059 (Olfactory 227)	11	10	90.91
001-005-060 (Olfactory 232)	101	93	92.08

001-005-061 (Olfactory 234)	12	6	50.00
001-005-062 (Olfactory 238)	12	11	91.67
001-005-063 (Olfactory 241)	49	36	73.47
001-005-064 (Olfactory 243)	17	10	58.82
001-005-065 (Olfactory 244)	18	13	72.22
001-005-066 (Olfactory 246)	35	33	94.29
001-005-067 (Olfactory 247)	40	33	82.50
001-005-068 (Olfactory 251)	18	17	94.44
001-005-069 (Olfactory 257)	19	11	57.89
001-005-070 (Olfactory 272)	14	4	28.57
001-005-071 (Olfactory 277)	21	14	66.67
001-005-072 (Olfactory 281)	13	11	84.62
001-005-073 (Olfactory 283)	17	8	47.06
001-005-074 (Olfactory 287)	17	16	94.12
001-005-075 (Olfactory 288)	24	23	95.83
001-005-076 (Olfactory 297)	13	6	46.15
001-005-077 (Olfactory 298)	10	5	50.00
001-005-078 (Olfactory 300)	12	8	66.67
001-005-079 (Olfactory 306)	15	13	86.67
001-005-080 (Olfactory 307)	12	7	58.33
001-005-081 (Olfactory 308)	37	28	75.68
001-005-082 (Olfactory 309)	29	28	96.55
001-005-083 (Olfactory 312)	16	10	62.50
001-005-084 (Olfactory 314)	28	19	67.86
001-005-085 (Olfactory 320)	18	8	44.44
Overall	2022	1465	72.45
001-007-001 (Adenosine)	68	65	95.59
001-007-002 (Purinoceptors)	127	124	97.64
Overall	195	189	96.92
001-008-001 (Gonadotropin-releasing hormone type I)	11	9	81.82
001-008-002 (Gonadotropin-releasing hormone type II)	36	34	94.44
001-008-003 (Adipokinetic hormone like)	17	14	82.35
Overall	64	57	89.06
001-009-001 (Thyrotropin-releasing hormone)	19	17	89.47
001-009-002 (Growth hormone secretagogue like)	10	10	100.00
Overall	29	27	93.10
001-016-001 (SREB)	10	10	100.00
001-016-002 (Mas proto-oncogene & Mas-related (MRGs))	106	104	98.11
001-016-003 (EBV-induced)	12	12	100.00
001-016-004 (LGR like)	38	38	100.00
001-016-005 (Cysteinyl leukotriene)	48	40	83.33
001-016-006 (Free fatty acid receptor 3)	18	18	100.00
001-016-007 (Class A Other)	111	106	95.50
Overall	343	328	95.63
Overall (of all sub-subfamilies)	4924	3954	80.30

Level 5 (**Subtype**, each sequence in a subtype has less than 90% identity with other sequences in the same subtype.)

Subtype No. (Name)	*Tol(i)*	*C(i)*	*ACC (%)*
001-001-001-001(Musc. acetylcholine Vertebrate type 2)	13	10	76.92
001-001-001-002(Musc. acetylcholine Vertebrate type 5)	15	12	80.00
001-001-001-003(Musc. acetylcholine Non Vertebrate)	38	37	97.37
Overall	66	59	89.39
001-001-002-001(Alpha Adrenoceptors)	143	139	97.20
001-001-002-002(Beta Adrenoceptors)	37	35	94.59
Overall	180	174	96.67
001-001-003-001(Dopamine Vertebrate type 1)	24	18	75.00
001-001-003-002(Dopamine Vertebrate type 2)	40	38	95.00
001-001-003-003(Dopamine Vertebrate type 4)	10	10	100.00
001-001-003-004(Dopamine Vertebrate type 5)	10	10	100.00
001-001-003-005(Dopamine Insect type 1)	18	16	88.89
001-001-003-006(Dopamine Insect type 2)	28	26	92.86
Overall	130	118	90.77
001-001-004-001(Histamine type 1)	13	12	92.31
001-001-004-002(Histamine type 2)	12	12	100.00
001-001-004-003(Histamine type 3)	39	34	87.18
001-001-004-004(Histamine type 4)	11	10	90.91
Overall	75	68	90.67
001-001-005-001(Serotonin 1)	61	44	72.13
001-001-005-002(Serotonin 2)	61	58	95.08
001-001-005-003(Serotonin type 4)	26	25	96.15
001-001-005-004(Serotonin 5)	15	15	100.00
001-001-005-005(Serotonin type 7)	32	24	75.00
001-001-005-006(Serotonin Insect)	34	32	94.12
Overall	229	198	86.46
001-001-006-001(Octopamine type 1)	10	10	100.00
001-001-006-002(Octopamine type 2)	11	10	90.91
001-001-006-003(Octopamine type 6)	11	11	100.00
Overall	32	31	96.88
001-001-007-001(Trace amine type 1)	14	13	92.86
001-001-007-002(Trace amine type 9)	18	17	94.44
001-001-007-003(Trace amine type 15)	17	17	100.00
001-001-007-004(Trace amine type 16)	43	42	97.67
Overall	92	89	96.74
001-002-002-001(Neuromedin B receptor)	25	21	84.00
001-002-002-002(Gastrin-releasing peptide receptor)	20	18	90.00
001-002-002-003(Bombesin type 4)	10	10	100.00
Overall	55	49	89.09
001-002-003-001(Bradykinin type B1)	13	13	100.00
001-002-003-002(Bradykinin type B2)	27	26	96.30
Overall	40	39	97.50
001-002-007-001(Interleukin-8 type A)	10	5	50.00
001-002-007-002(Interleukin-8 type B)	23	19	82.61
Overall	33	24	72.73
001-002-008-001(C-C Chemokine)	174	172	98.85
001-002-008-002(C-X-C Chemokine)	44	40	90.91
001-002-008-003(C-X3-C Chemokine)	10	10	100.00
001-002-008-004(XC Chemokine)	15	14	93.33
Overall	243	236	97.12

001-002-010-001(Endothelin type A)	11	11	100.00
001-002-010-002(Endothelin type B)	22	22	100.00
Overall	33	33	100.00
001-002-011-001(Melanocortin type 1)	27	27	100.00
001-002-011-002(Melanocortin type 3)	11	11	100.00
001-002-011-003(Melanocortin type 5)	19	17	89.47
Overall	57	55	96.49
001-002-013-001(Neuropeptide Y type 1)	20	14	70.00
001-002-013-002(Neuropeptide Y type 2)	29	24	82.76
001-002-013-003(Neuropeptide Y type 4)	11	11	100.00
001-002-013-004(Neuropeptide Y type 5)	10	10	100.00
001-002-013-005(Neuropeptide Y / peptide YY)	32	27	84.38
001-002-013-006(Neuropeptide FF type 2)	10	7	70.00
001-002-013-007(Orexigenic neuropeptide QRFP)	14	10	71.43
001-002-013-008(GPR74 like)	133	117	87.97
001-002-013-009(GPR83 like)	17	13	76.47
Overall	276	233	84.42
001-002-015-001(Opioid type K)	10	10	100.00
001-002-015-002(Opioid type M)	22	22	100.00
001-002-015-003(Opioid type X)	12	12	100.00
Overall	33	33	100.00
001-002-016-001(Somatostatin type 1)	12	9	75.00
001-002-016-002(Somatostatin type 2)	19	12	63.16
001-002-016-003(Somatostatin type 3)	14	11	78.57
001-002-016-004(Somatostatin type 5)	45	36	80.00
Overall	90	68	75.56
001-002-017-001(Substance P (NK1))	26	23	88.46
001-002-017-002(Substance P (NK2))	11	10	90.91
001-002-017-003(Tachykinin like 2)	39	35	89.74
Overall	76	68	89.47
001-002-018-001(Vasopressin)	20	19	95.00
001-002-018-002(Vasopressin type 2)	13	11	84.62
001-002-018-003(Oxytocin)	10	10	100.00
001-002-018-004(Vasotocin)	54	53	98.15
Overall	97	93	95.88
001-002-019-001(Galanin type 1)	25	23	92.00
001-002-019-002(Allostatin)	40	38	95.00
001-002-019-003(Kiss receptor (GPR54))	45	41	91.11
Overall	110	102	92.73
001-002-020-001(Proteinase-activated type 1)	18	18	100.00
001-002-020-002(Proteinase-activated type 2)	37	36	97.30
Overall	55	54	98.18
001-002-024-001(Neuromedin U)	32	29	90.63
001-002-024-002(PRXamide)	15	14	93.33
001-002-024-003(PRXamide and Pyrokinin like)	25	24	96.00
Overall	72	67	93.06
001-004-001-001(Vertebrate blue/green opsin)	113	109	96.46
001-004-001-002(Vertebrate red opsin)	28	25	89.29
001-004-001-003(Vertebrate short wavelength opsin)	34	34	100.00
001-004-001-004(Vertebrate opsin type 3)	11	10	90.91
001-004-001-005(Vertebrate opsin type 5)	19	15	78.95
001-004-001-006(Vertebrate melanopsin)	26	26	92.86
001-004-001-007(Vertebrate Peropsin)	12	10	83.33
Overall	245	229	93.47

001-004-002-001(Rhodopsin Arthropod short wavelenght)	137	136	99.27
001-004-002-002(Putative Arthropod opsin)	18	18	100.00
Overall	155	154	99.35
001-007-001-001(Adenosine type 1)	18	18	100.00
001-007-001-002(Adenosine type 2)	55	53	96.36
001-007-001-003(Adenosine type 3)	12	10	83.33
Overall	85	81	95.29
001-007-002-001(Purinoceptor P2RY1-4,6,11 GPR91)	77	76	98.70
001-007-002-002(P2RY5,8,9,10 GPR23,35,92,174)	70	66	94.29
001-007-002-003(Purinoceptor P2RY12-14 GPR87 (UDP-Glucose))	35	34	97.14
Overall	182	176	96.70
Overall (of all subtypes)	2741	2531	92.34
